# Characterization and Botanical Differentiation of Monofloral and Multifloral Honeys Produced in Cyprus, Greece, and Egypt Using Physicochemical Parameter Analysis and Mineral Content in Conjunction with Supervised Statistical Techniques

**DOI:** 10.1155/2018/7698251

**Published:** 2018-06-05

**Authors:** Ioannis K. Karabagias, Artemis P. Louppis, Stavros Kontakos, Chryssoula Drouza, Chara Papastephanou

**Affiliations:** ^1^Laboratory of Food Chemistry, Department of Chemistry, University of Ioannina, Ioannina 45110, Greece; ^2^cp Foodlab Ltd., Polifonti 25, 2047 Strovolos, Nicosia, Cyprus; ^3^Department of Social Administration and Political Science, Democritus University of Thrace, Komotini 69100, Greece; ^4^Department of Agricultural Sciences, Biotechnology and Food Science, Cyprus University of Technology, 3036 Limassol, Cyprus

## Abstract

Thirty-four honey samples donated by beekeepers and purchased from supermarkets were collected during harvesting years 2010–2014 from Cyprus, Greece, and Egypt. The aims of this study were to characterize honey samples and, if possible, to differentiate honeys according to the honey type on the basis of physicochemical parameter values, mineral content, and their combination using supervised statistical techniques (linear discriminant analysis (LDA)). Physicochemical parameters (colour, pH, free acidity, total dissolved solids, salinity, electrical conductivity, and moisture content) were determined according to official methods, while minerals (Al, As, B, Ba, Be, Ca, Cd, Co, Cr, Cu, Fe, Hg, Mg, Mn, Mo, Ni, P, Pb, Sb, Si, Ti, Tl, V, and Zn) using inductively coupled plasma optical emission spectrometry. The majority of honey samples analyzed met the quality criteria set by the European directive and national decision related to honey. Implementation of multivariate analysis of variance (MANOVA) and LDA on specific physicochemical parameters, minerals, or their combination provided a satisfactory classification of honeys according to floral type. The overall correct classification rate (based on the cross-validation method) was 79.4% using 7 minerals and 91.2% using 8 physicochemical parameters. When the 15 parameters were combined, the classification rate of Egyptian honeys was improved by 25%.

## 1. Introduction

The ability to determine the origins of honey, the product of *Apis mellifera* honeybees, has become very important for protection of both consumers and beekeepers, as certain types of honey and their place of origin can impact the pricing, which can lead to counterfeits being produced to increase the value of a lesser product.

In that sense, terms such as protected designation of origin (PDO), protected geographical indication (PGI), or traditional specially guaranteed (TSG) [1] have been set to highlight several products' quality produced in different parts of the world, including honey. Some typical examples involve the Spanish PDO “Miel de Granada,” the Greek fir tree honey from “Menalon,” and the pine-thyme honey (Pefkothymaromelo) from Crete [2–4]. Such products have the potential to attract consumers, producers, and exporters, and they create a basis for different types of studies, that is, economic studies or studies related to authenticity, using a data set of several physicochemical parameters and chemometric analyses [5, 6].

The usual method to determine the botanical origin of honey is pollen recognition (melissopalynology). However, this approach has certain limitations such as pollen counting procedure and identification, interpretation of results which is difficult and requires trained analysts, considerable time of analysis, or limited geographical origin determination applications [7]. Hence, the combination of pollen analysis with physicochemical and sensory characteristics can overcome the limitations of pollen analysis [8].

On the contrary, the great variability in honey chemical composition is closely related to botanical and geographical origins with regard to soil, specific vegetation, climate conditions, bee species, processing technologies, and honey extraction methods. Thus, these variations, that is, in the mineral content and/or physicochemical parameters of honey, offer a great opportunity for research related to its botanical or geographical discrimination (authentication), using supervised statistical techniques [9, 10].

Besides, in recent years, there has been a rising presence of monofloral honeys in markets, more expensive than multifloral ones, so possible adulteration by honey mixtures must be controlled.

Therefore, the objectives of the present study were to provide information about conventional physicochemical parameters and mineral content of Cypriot, Greek, and Egyptian honeys (which are categorized as multifloral and unifloral honeys) and, if possible, to differentiate these honeys according to the floral type, using a set of specific conventional physicochemical parameters, minerals, or their combination in conjunction with modern and supervised statistical techniques by partial use of melissopalynological data.

To our knowledge, data for the selected physicochemical parameters or minerals have not been reported previously [2–7, 9–13] for these specific honey types, and this constitutes the novelty of the present work.

## 2. Materials and Methods

### 2.1. Honey Samples

A total of 34 honey samples were collected during the harvesting periods 2011-2012 from Cyprus, 2010–2012 from Greece, and 2013-2014 from Egypt (Table 1).

Cypriot honeys were supplied by local professional beekeepers from the three different regions: Larnaca, Limassol, and Nicosia (14 multifloral honey samples). According to their declaration, honey samples were multifloral of specific vegetation grown in Cyprus. In particular, honeys from Larnaca consisted of *Acacia*, *Eucalyptus*, *Phoenix*, *Albizia*, rosemary, *Heliotropium europaeum*, *Parkinsonia aculeata*, and *Thymus* spp. Honeys from Limassol consisted of wild rosemary (*Teucrium creticum*), *Rhus coriaria*, *Onopordum acanthium*, *Thymus* spp., *Heliotropium europaeum*, *Eucalyptus*, *Ceratonia*, and *Opuntia ficus-indica*. Finally, honeys from Nicosia consisted of *Rhus coriaria*, *Thymus* spp., wild flowers, *Eucalyptus*, *Teucrium creticum*, *Heliotropium europaeum*, *Parkinsonia aculeata*, and *Phoenix*.

Honey samples from Greece were supplied by professional beekeepers from seven regions: Messinia (1 fir) (*Abies cephalonica*), Lakonia (1 wild thyme) (*Thymus serpyllum*), Rhodes (2 pines and 1 thyme) (*Pinus* and *Thymus* spp., resp.), Arta (2 citrus + erica) (*Citrus* and *Erica* spp.), Ioannina (2 multiflorals), Karditsa (1 fir) (*Abies cephalonica*), Metsovo (1 fir) (*Abies cephalonica*) (1 multifloral), and Thassos (1 pine) (*Pinus* spp.). As in the case of Cypriot honeys, the botanical origin of Greek honey samples was confirmed by the beekeepers.

Finally, Egyptian honeys (8 samples) were purchased from the greater Cairo area. Melissopalynological analysis was carried out according to a previous study [6] for the Egyptian honey samples, since these were purchased from supermarkets. Respective label description was Vitrac pure natural clover honey produced by HERO NFI in Egypt. Results showed that seven of the eight honey samples were classified as clover (*Trifolium alexandrinum*). The percentage of *Trifolium* pollen that was considered as predominant pollen was set at >45%. The percentage of *Trifolium* pollen grains varied between 41 and 91% [14]. All honey samples were stored in dark-coloured glass containers, shipped to the laboratory, and maintained at 4°C until analysis. Each batch of samples was analyzed within the first month of the same harvesting year of collection.

### 2.2. Reagents and Solutions

All chemicals used in the present study were of analytical grade [10].

### 2.3. Preparation of Honey Samples for Inductively Coupled Plasma Optical Emission Spectrometry (ICP-OES) Analysis: Instrumentation and Conditions

Preparation of honey samples for ICP-OES analysis and ICP-OES instrumentation and conditions are given in a previous study [10]. Twenty-four minerals were determined including Al, As, B, Ba, Be, Ca, Cd, Co, Cr, Cu, Fe, Hg, Mg, Mn, Mo, Ni, P, Pb, Sb, Si, Ti, Tl, V, and Zn. The emission wavelengths (nm) for all minerals are given in a previous study [10]. The emission wavelength for P was 178.3 nm.

### 2.4. Physicochemical Parameters

The following physicochemical parameters were determined according to the harmonized methods of the International Honey Commission: pH, moisture content, electrical conductivity, and free acidity [15]. For the determination of honey moisture content, an ATC refractometer (Bellingham + Stanley, UK), covering the range of moisture between 12 and 27%, was used. The interlaboratory variation, determined as the coefficient of variation of independent measurements (RSD_R_ %), is the quality parameter most often used to compare the precision of a developed analytical method. RSD_R_ % was calculated as

(1) $$\begin{array}{c} 100\;\times \;\left(\frac{R}{X}\right)\times 2.8, \end{array}$$

where *R* is the reproducibility value of independent measurements and *X* is the average of carrying determinations [15]. The results reported are the mean of three independent determinations (*n*=3).

### 2.5. Colour Parameters *L*^*∗*^, *a*^*∗*^, and *b*^*∗*^

The honey surface colour was measured according to the method proposed by Karabagias et al. [6]. Before making measurements, honey solutions were filtered for removal of any coarse particles. The precision of the analytical procedure followed was estimated by calculating RSD_R_ % values. The results reported are the mean of five independent determinations (*n*=5).

### 2.6. Salinity and Total Dissolved Solids (TDS)

Salinity and total dissolved solids of a 20% (w/v) honey solution in distilled water were measured at 20°C using a Delta OHM, model HD 3456.2, conductivity meter (Padova, Italy) with 4-ring and 2-ring conductivity/temperature probes. Temperature was measured by 4-wire Pt100 and 2-wire Pt1000 sensors by immersion. The probe was calibrated automatically resorting to the 1413 *μ*S/cm conductivity standard solution (Hanna Instruments, Inc., Woonsocket, USA). Results were expressed as g/L and mg/L, respectively. The precision of the analytical procedure followed was estimated by calculating RSD_R_ % values. The results reported are the mean of three independent determinations (*n*=3).

### 2.7. Statistical Analysis

All data were subjected to statistical analysis using the SPSS statistics software (v.20.0, SPSS Inc., 2012) by application of multivariate analysis of variance (MANOVA) and linear discriminant analysis (LDA).

Comparison of the means was achieved by MANOVA in order to determine those significant physicochemical parameter or mineral content values that could aid in the geographical differentiation of Cypriot, Greek, and Egyptian honeys. Pillai's trace and Wilks' lambda indices were computed, as inverse quality criteria, to determine a possible significant effect of physicochemical parameter and mineral content values on the geographical origin of honeys. The homogeneity of variability was tested by application of Box's M index, since the number of the collected honey samples from each region was different. Both original and leave-one-out cross-validation methods were used to test the correct classification ability. The cross-validation method provides an additional verification of the developed model of discrimination. In most cases, the correct prediction rates obtained by using the cross-validation method are lower compared to those obtained by the original method but are more reliable [10]. Correlations were obtained by Pearson's correlation coefficient (*r*) at the confidence level *p* < 0.05.

## 3. Results and Discussion

### 3.1. Validity of the Methods Used for the Determination of Physicochemical Parameters and Minerals

#### 3.1.1. Physicochemical Parameters

The methods used for the measurements of pH, moisture content, electrical conductivity, free acidity, total dissolved solids, salinity, and the colour parameters *L*^*∗*^, *a*^*∗*^, and *b*^*∗*^ showed different coefficient variation values (RSD_R_ %) depending on the honey matrix and the nature of analysis (Table 1). However, RSD_R_ % values of moisture content, electrical conductivity, and free acidity are in conformity to the International Honey Commission guidelines regarding the precision of the analytical method followed for the determination of honey conventional physicochemical parameters [15]. Regarding the determination of physicochemical parameters that are not regulated, that is, colour parameters (especially *L*^*∗*^ and *b*^*∗*^), pH, total dissolved solids, and salinity, the method developed showed RSD_R_ % <10% (Table 1).

#### 3.1.2. Minerals

*(1) Calibration Curves and Linearity*. The calibration curves were prepared using 8 data points: 50, 100, 150, 200, 250, 300, 350, and 400 *μ*g/kg of the mineral standard solution (ICP multielement standard solution XVI 1.09487.0100 of 100 mL volume and 100 mg/L concentration; Merck, Darmstadt, Germany). The correlation coefficient (*R*^2^) was found to be >0.993 for the regression equation obtained for each mineral that was determined in the present study.

*(2) Precision*. The repeatability of the sample application and measurement of the peak area of each mineral was evaluated using three replicates of the standard (100 mg/L) on the same day and over three days (three replicates each day). The intra- and interday variations were estimated by relative standard deviation values which were lower than 5% for each analyzed mineral with respect to honey botanical origin (different honey matrixes) (Table 2).

*(3) Limit of Detection (LOD) and Limit of Quantification (LOQ)*. The limit of detection (LOD) and limit of quantification (LOQ) were estimated by spiking a blank sample (ultrapure water obtained from Milli-Q, Millipore, Bedford, MA, USA) three times with the standard mineral solution at low concentrations, and the signal-to-noise ratio was determined. The LOD was defined as 3 : 1 and the LOQ as 10 : 1. The LOD and LOQ values for each mineral are given in Table 2.

### 3.2. Physicochemical Parameters of Cypriot, Greek, and Egyptian Honeys

A high moisture content in honey (>20 g/100 g) is undesirable because it leads to fermentation during storage. Present moisture content values are within the limits (≤20%) set by the EU [8] for commercial honey samples. Multifloral honeys from Ioannina and Metsovo (Greece) showed variation in moisture content depending on botanical and geographical origins. In addition, moisture content values for fir honeys collected from Messinia, Karditsa, and Metsovo (Greece) were in accordance (≤18.5%) with the Greek decision [16] involving the botanical identification of monofloral honeys. The lower moisture values were recorded for Cypriot honeys, indicative of the climatic conditions and floral type of honeys in this area. Pure clover labeled honey recorded an average value of moisture, slightly higher than that of Greek honeys. However, it should not be forgotten that variations in moisture content, among different honey types and geographical origins, have been reported previously for Turkish [9], Spanish [11, 17], and Greek honeys [6, 11].

Honey samples from Cyprus had lower pH values as compared to Greek or Egyptian honeys since these were multifloral honeys. pH in honey has been reported to range between 3.20 and 4.50 indicating a natural acidity. Present pH values for Greek honeys are in accordance with the previous work in the literature regarding Spanish [17], while Cypriot and Egyptian honeys recorded pH values in agreement with those reported for Malaysian honeys [18].

All honey samples analyzed had FA values below the upper limit (<50 meq/kg) set by the EU [8]. Present FA values are within the range reported previously for Spanish [11, 17], Moroccan [19], and Greek commercial honeys [6, 11]. The higher FA values were recorded for honey samples from Greece. Clover honeys from Egypt recorded pH values in excellent conformity with citrus honeys from Egypt, as shown in the previous work [11].

Electrical conductivity (EC) is a conventional physicochemical parameter related to the botanical origin of honey, and it is used very often in routine honey quality control. According to the EU [8], the maximum limit value of EC for thyme honeys is 0.80 mS/cm, while honeydew honeys often have EC ≥0.80 mS/cm. The Greek decision for monofloral honeys [16] sets the EC values for pine, fir, thyme, and citrus honeys to be ≥0.90, ≥1.0, ≤0.60, and ≤0.45, respectively. Based on the aforementioned directives, pine honey-labeled samples from Rhodes could not be classified as monofloral pine honeys. Mixed citrus honeys from Arta (citrus + erica) recorded EC values slightly greater than 0.60 mS/cm, indicating the correct information regarding the mixed honey botanical origin provided by the beekeepers. Cypriot honeys recorded typical EC values for blossom honeys according to the European directive and Greek decision related to honey origin [8, 16] (Table 1). The variations of EC values among honey samples analyzed are in agreement with previous studies involving Greek, Spanish, Malaysian, Moroccan, and Sardinian honeys of different botanical and geographical origins [6, 11, 17–21].

Total dissolved solids (TDS) is a measure of the combined content of all inorganic and organic substances present in honey in molecular, ionized, or microgranular (colloidal solution) suspended forms. The TDS content of Greek honeys was higher than that of Egyptian and Cypriot honeys. An excellent Pearson's correlation (*r*) between EC and TDS was obtained, according to the botanical origin of commercial honeys (*r*=0.995), indicating that the salts dissolved in honey are the carriers of EC. This is in agreement with the previous work in the literature involving Algerian honeys [22]. To the best of our knowledge, data on TDS content have never been reported before for Cypriot, Greek, or Egyptian honeys.

Salinity is the saltiness or dissolved salt content in an aqueous medium. In general, honeys from Greece recorded higher salinity values as compared to those from Cyprus or Egypt. Additionally, a very strong Pearson's correlation (*r*) was recorded between EC and salinity (*r*=0.927), according to the botanical origin of honeys. Therefore, salinity and TDS content are proposed as supplementary physicochemical markers for the botanical origin differentiation of honey.

Honey colour may vary with respect to several parameters like Maillard reaction products, fructose caramelization, botanical origin, pollen content, and polyphenol or mineral content [18]. Colour parameters (*L*^*∗*^ and *b*^*∗*^) varied significantly according to the different botanical origins grouped in the tested geographical zones. In particular, Cypriot and Egyptian honeys were the brightest (higher *L*^*∗*^ values) compared to Greek honeys, whereas Greek honeys possessed numerous green and yellow pigments (higher *a*^*∗*^ and *b*^*∗*^ values, resp.) (Table 2). This is in agreement with the previous work in the literature, regarding Turkish [5], Greek [6], and Spanish [17] honeys. It should also be mentioned that, in a recent study, citrus honeys from Egypt proved to have similar brightness [11] with Egyptian clover honeys of the present study. However, respective comparison of *a*^*∗*^ and *b*^*∗*^ values indicates substantial differences, originating from the presence of different pigments.

### 3.3. Mineral Content of Cypriot, Greek, and Egyptian Honeys

The mineral content (mg/kg) of Cypriot, Greek, and Egyptian honeys is given in Table 2. Total mineral content (the sum of individual minerals) followed the order Greece > Cyprus > Egypt. The most dominant minerals were Ca, P, Si, and Mg followed by Zn, Al, B, Fe, and Mn. The higher, in general, mineral content (P, Mg, Si, Fe, and Mn) in honey samples from Greece, as compared to those from Cyprus, is attributed to the presence of honeydew, pine, and fir honey samples (dark-coloured honeys) being collected from these regions. It should also be stressed that the content of Mg, Zn, Al, and Mn of honey samples of the present study was higher compared to that in the previous work involving Egyptian, Spanish, Moroccan, and Greek citrus honeys (light-blossom honeys) [11]. It has been documented previously that dark-coloured honeys possess higher mineral content, as compared to light-blossom honeys [9, 11, 20]. An exception to this was the Ca content of multifloral honeys from Cyprus and clover honeys from Egypt, which was higher compared to that of Greek honeys. This is probably owed to the specific soil conditions in these regions including the vegetation grown. In the recent work [11], the Ca content of Egyptian citrus honeys was lower compared to present results, indicating the strong impact of botanical origin on honey mineral content.

Furthermore, the higher P content (mg/kg) was recorded in wild thyme honey sample no. 4 from Lakonia and mixed citrus honey (citrus + erica) no. 10 from Arta. This indicates the effectiveness of both botanical origin (i.e., the presence of honeydew elements in the wild thyme honey sample) and geographical origin (i.e., certain soil conditions in Lakonia and Arta) to the overall mineral content of honey. This is in very good agreement with the previous work in the literature [20]. Further comparison of present results, regarding P content, with citrus honeys produced in Egypt, Spain, Morocco, and Greece will totally reveal significant differences [11], as in the honey samples analyzed P recorded much higher values (mg/kg) (Table 2).

In addition, small amounts of As (<0.08 mg/kg), Ba (<0.06 mg/kg), Be (<0.06 mg/kg), Cd (<0.05 mg/kg), Co (<0.03 mg/kg), Cr (<0.12 mg/kg), Cu (<0.35 mg/kg), Hg (<0.03 mg/kg), Mo (<0.08 mg/kg), Ni (<0.21 mg/kg), Pb (<0.08 mg/kg), Sb (<0.14 mg/kg), Ti (<0.09 mg/kg), Tl (<0.20 mg/kg), and V (<0.11 mg/kg) were determined in all honey samples analyzed. The level of these minerals is in very good agreement with the previous work in the literature involving Spanish [9, 24], Greek [10, 11], Egyptian [11], Malaysian [18], and Swiss [25] honeys. These small amounts (<1 mg/kg) of toxic heavy metals like Cd and Pb give an additional quality standard to the honey samples analyzed.

Finally, a very strong Pearson's correlation (*r*=0.983) was obtained between average values of EC and total mineral content with respect to the specific honey types collected from Cyprus, Greece, and Egypt. This observation shows that the electrical conductivity of honey is owed (among others) to charged compounds such as minerals and highlights at a greater degree the reliability of the two independent methodologies followed in the present study: conventional physicochemical parameter analysis (for the EC determination) and ICP-OES analysis (for the determination of minerals).

### 3.4. Classification of Cypriot, Greek, and Egyptian Honeys according to Botanical Origin Based on Physicochemical Parameter Values

Pillai's trace = 1.600 (*F*=10.659; *p* value = 0.000) and Wilks' lambda = 0.037 (*F*=10.783; *p* value = 0.000) indices pointed out the existence of a significant effect of the botanical origin (independent variables) on physicochemical parameter values (dependent variables) of the 34 honey samples. Eight of the 9 physicochemical parameters determined were found to be significant (*p* < 0.05) for the differentiation of honeys (Table 3). Thus, these 8 parameters were subjected to LDA. The results showed that two statistically significant discriminant functions were formed: Wilks' lambda = 0.044, *X*^2^ = 86.010, df = 16, and *p* value = 0.000 for the first function and Wilks' lambda = 0.308, *X*^2^ = 32.403, df = 7, and *p* value = 0.000 for the second. Testing of the uniformity of variability (Box's M index) was not significant at the 95% confidence level (*p* value = 0.052), showing the existence of uniformity of sample variability for honeys collected from the three regions. The first discriminant function accounted for 72.8% of the total variance, while the second accounted for 26.2%. Both accounted for 99.0% of the total variance, considered an excellent rate.

**Figure 1:**
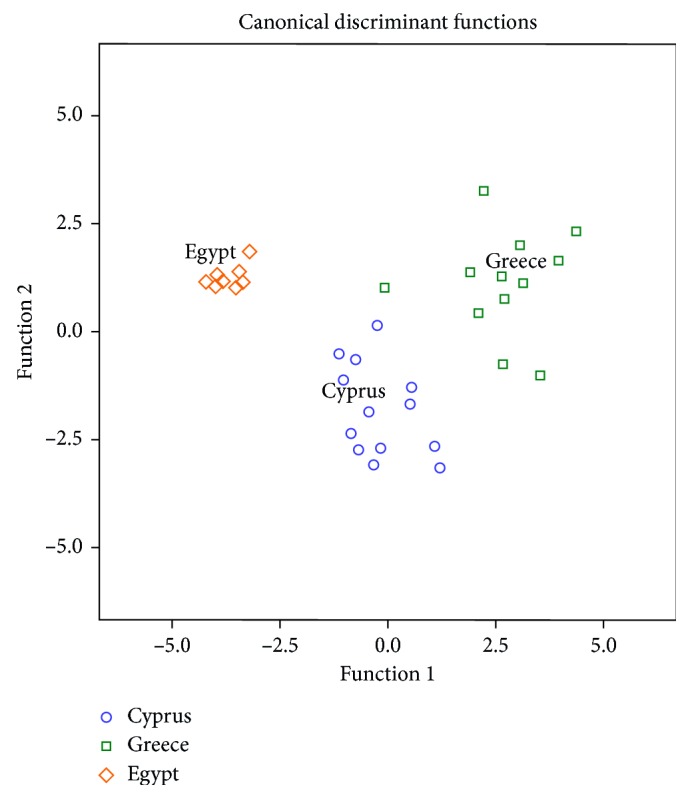
Differentiation of Greek, Cypriot, and Egyptian honeys according to botanical origin based on 8 physicochemical parameters.

In Figure 1, it is shown that honeys from Cyprus, Greece, and Egypt are well separated. The first discriminant function differentiates honey samples from Greece, while the second differentiates those from Egypt. The physicochemical parameters that contributed to the first and the second discriminant functions are given in Table 3. The overall correct classification rate was 97.1% for the original method and 91.2% for the cross-validation method, the latter being considered very satisfactory for this method. In particular, the correct classification rate was 100%, 92.3%, and 100% for honeys from Cyprus, Greece, and Egypt using the original method and 100%, 76.9%, and 100% for honeys from Cyprus, Greece, and Egypt, respectively, by using the cross-validation method.

Present results are in agreement with previous works in the literature, in which the use of certain physicochemical parameters (i.e., free acidity, pH, electrical conductivity, ash, hydroxymethylfurfural, diastase activity, and colour attributes) proved to be an effective tool for the geographical and botanical discrimination of several honey types produced in different parts of the world [6, 11, 17, 18, 20].

### 3.5. Classification of Cypriot, Greek, and Egyptian Honeys according to Botanical Origin Based on Mineral Content Analysis

The next step was to investigate whether minerals could provide a higher classification rate. The 34 honey samples were subjected to MANOVA to determine which minerals were significant for the differentiation of honeys from the three different regions. Dependent variables included the 9 minerals, while botanical origin was taken as the independent variable. Pillai's trace = 1.401 (*F*=6.235; *p* value = 0.001) and Wilks' lambda = 0.065 (*F*=7.470; *p* value = 0.001) index values showed the existence of a significant multivariable effect of botanical origin on honey's mineral content. Seven of the 9 minerals (Table 3) were found to be significant (*p* < 0.05) for the differentiation of honeys. Thus, these 7 minerals were subjected to LDA.

Results showed that two statistically significant discriminant functions were formed: Wilks' lambda = 0.083, *X*^2^ = 69.586, df = 14, and *p* value = 0.001 for the first function and Wilks' lambda = 0.518, *X*^2^ = 18.437, df = 6, and *p* value = 0.002 for the second. As in the case of physicochemical parameters, testing of the uniformity of variability (Box's M index) was insignificant at the 95% confidence level showing the existence of uniformity of sample variability for each botanical origin. The first discriminant function accounted for 86.8% of the total variance, while the second accounted for 13.9%. Both accounted for 98.7% of the total variance, which is also considered an excellent rate.

**Figure 2:**
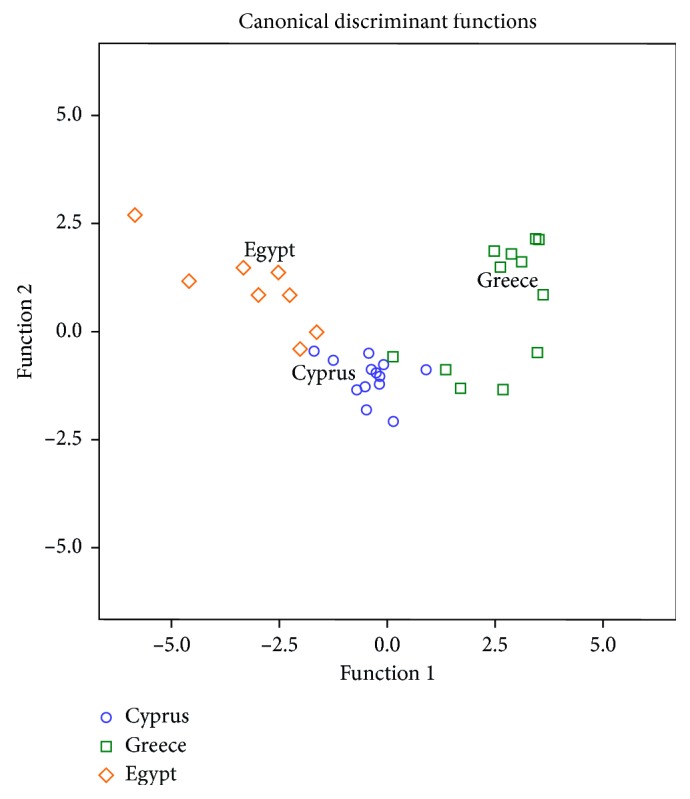
Differentiation of Greek, Cypriot, and Egyptian honeys according to botanical origin based on 7 minerals.

In Figure 2, it is shown that honeys from all the tested regions are clearly separated based on the first and second discriminant functions. The first discriminant function differentiates honeys from Greece and Egypt, whereas the second discriminant function differentiates honeys from Cyprus. The overall correct classification rate was 84.8% using the original method and 79.4% using the cross-validation method, a satisfactory value especially for the second method. More specifically, the original method classified correctly honeys from Cyprus, Greece, and Egypt by 100%, 77%, and 75%, respectively. In the case of the cross-validation method, the respective classification rates were 92.3%, 69.2%, and 75%. The use of certain minerals has the potential to provide accurate classification rates for several honey types produced in different parts of the world for both geographical and botanical origin determination of honey [10, 11, 23–26].

As it can be observed, minerals provided a much lower classification rate as compared to the physicochemical parameters. This finding is in accordance with a recent work in the literature [10, 11] but in contrast to previous works [24, 26], in which the classification rate of Spanish thyme, eucalyptus, orange blossom, rosemary, and heather honeys according to the production area and botanical origin, based on certain minerals and using discriminant analysis, was approx. 100%. Differences in the overall classification rate reported in previous works compared to results of the present study may be attributed to several factors such as botanical origin of honeys, similarities in soil and vegetation conditions in honey production areas, harvesting year, honey processing and extraction methods, the analytical methodology applied, beekeepers' practices/handling, chemometric techniques applied, or any other unpredicted external factor. For example, when Fernández-Torres et al. [26] used certain minerals and principal component analysis to classify Spanish eucalyptus, orange blossom, rosemary, and heather honeys according to the floral type, the classification rate based on the two principal components was 71.59%, best differentiating heather and eucalyptus honeys.

Based on the aforementioned results, the next step was to investigate whether the combination of the significant physicochemical parameters and minerals (*p* < 0.05) could provide a higher classification rate as compared to minerals alone or to improve the classification rate of a specific honey type.

### 3.6. Classification of Cypriot, Greek, and Egyptian Honeys according to Botanical Origin Based on the Combination of Physicochemical Parameter and Mineral Content Values

Pillai's trace = 1.782 (*F*=6.799; *p* value = 0.000) and Wilks' lambda = 0.007 (*F*=8.798; *p* value = 0.000) index values showed that there was a significant multivariable effect of botanical origin on mineral/physicochemical parameter values. Seven minerals (B, Ca, Si, Fe, P, Mn, and Mg) and 8 physicochemical parameter values (pH, electrical conductivity, moisture, free acidity, total dissolved solids, salinity, *L*^*∗*^, and *b*^*∗*^) served as the significant variables (*p* < 0.05) for the differentiation of honeys. Thus, the combined values of 7 minerals and 8 physicochemical parameters were subjected to LDA.

The results showed that two statistically significant discriminant functions were formed: Wilks' lambda = 0.009, *X*^2^ = 110.727, df = 32, and *p* value = 0.000 for the first function and Wilks' lambda = 0.244, *X*^2^ = 33.106, df = 15, and *p* value = 0.005 for the second.

**Figure 3:**
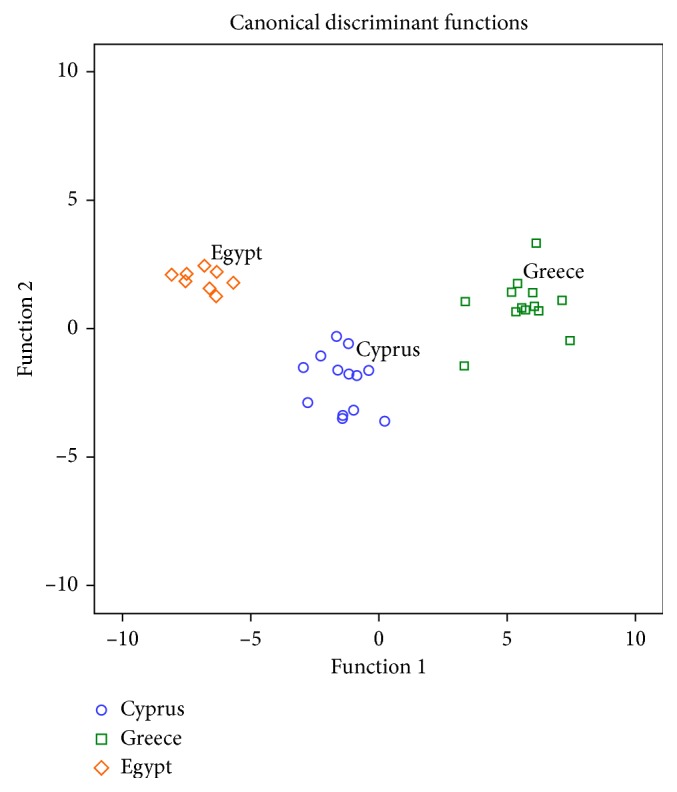
Differentiation of Greek, Cypriot, and Egyptian honeys according to botanical origin based on the combination of 8 physicochemical parameters and 7 minerals.

The first discriminant function accounted for 89.4% of the total variance, while the second accounted for 9.6%. Both accounted for 99% of the total variance. In Figure 3, it is shown that honeys from the investigated regions are clearly separated. The first discriminant function separates honeys from Greece as compared to those from Egypt, while the second discriminant function separates Cypriot honeys. The overall correct classification rate was 100% for the original method for all honey samples and 79.4% for the cross-validation method, a satisfactory value in general for the cross-validation method. The physicochemical parameters and minerals that contributed to the first and the second discriminant functions along with the discriminant function coefficients are given in Table 3. The physicochemical parameters and minerals that were strong markers of the provenience of the studied honeys are those with a largest absolute correlation between each variable and any discriminant function and are indicated with a symbol (Table 3).

As it can be observed, the combination of different physicochemical parameters and minerals did not increase the overall correct classification rate based on the cross-validation method. Therefore, it was not an effective approach in the present study for honey botanical origin differentiation in contrast to the previous work [5]. However, it should be highlighted that the distribution of honey samples in Figure 3 is greatly improved compared to that of minerals (Figure 2). At this point, to avoid any kind of misleading, it should be stressed that the correct classification rate obtained by the original method reflects the distribution of honey samples (Figures 1–3). The correct classification rate was 100% for all the investigated honeys using the original method and 84.6%, 61.6%, and 100% for honeys from Cyprus, Greece, and Egypt, respectively, by using the cross-validation method. The combination of minerals and physicochemical parameters improved only the classification rate of Egyptian honeys by 25% compared to that provided by minerals alone.

## 4. Conclusion

Botanical origin differentiation of honey produced in a specific country is of great importance for the characterization of “honey uniqueness” which may reflect its price in the global market. Physicochemical parameter analysis, including colour, may accurately provide information about honey origin in cases where melissopalynological analysis cannot be applied. On the contrary, minerals may assist in the nutritional characterization of honey and give additional information about its quality, floral type, and place of origin. The application of physicochemical parameter analysis provided a significantly higher discrimination rate (91.2%) of honeys according to botanical origin, compared to that of minerals (79.4%). However, an overall correct classification rate of ca. 80% using the cross-validation method may be considered satisfactory taking into account the (i) mixed floral honey types investigated, (ii) different harvesting years of honey collection, and (iii) different production countries.

## Figures and Tables

**Table 1:** Physicochemical parameters of Cypriot, Greek, and Egyptian honeys.

BO	GO	*L* ^*∗*^	*a* ^*∗*^	*b* ^*∗*^	pH	FA (meq/kg)	TDS (mg/L)	Salinity (g/L)	EC (mS/cm)	Moisture (g/100 g)
	*Cyprus*									
MF	Larnaca	73.72	−4.82	12.80	3.49	14.13	301.00	0.29	0.55	14.10
MF	Larnaca	71.04	−6.27	22.98	3.86	35.01	211.52	0.52	0.37	13.10
MF	Larnaca	71.49	−4.47	15.20	3.91	20.36	235.00	0.42	0.37	16.50
MF	Larnaca	71.69	−5.12	18.32	3.83	22.60	154.70	0.38	0.28	12.50
MF	Limassol	74.63	−4.84	5.20	3.64	12.64	284.00	0.29	0.54	13.45
MF	Limassol	72.16	−4.89	16.77	3.72	26.84	323.00	0.34	0.61	13.70
MF	Limassol	72.59	−4.77	14.76	3.70	21.40	150.50	0.38	0.28	13.40
MF	Limassol	73.48	−5.11	12.66	3.59	19.55	273.00	0.27	0.51	12.50
MF	Limassol	72.50	−3.64	21.49	3.65	29.32	167.80	0.40	0.32	16.10
MF	Limassol	72.92	−5.09	13.91	3.58	47.04	245.00	0.24	0.42	14.05
MF	Nicosia	74.24	−4.95	11.20	3.82	21.48	162.70	0.41	**0.32**	12.10
MF	Nicosia	71.50	−5.01	19.01	3.82	29.02	240.00	0.26	0.46	13.10
MF	Nicosia	75.77	−3.75	10.99	3.87	18.96	112.48	0.48	0.18	12.80
	Mean	72.90^a^	−4.83^d^	15.02^f^	3.73^i^	24.49^l^	220.05^n^	0.36^q^	0.40^t^	13.65^w^
	±SD	1.41	0.65	4.78	0.13	9.18	65.87	0.09	0.13	1.32
	RSD_R_ %	0.58	14.49	3.73	0.75	11.43	1.27	7.77	3.50	1.03

	*Greece*									
P	Rhodes	66.47	−4.70	32.68	4.24	22.00	404.00	0.41	0.78	15.50
F	Messinia	69.66	−5.73	26.84	5.05	25.00	346.00	0.54	1.01	17.60
P	Rhodes	66.53	−4.76	32.68	4.39	23.20	425.00	0.46	0.79	15.30
WT	Lakonia	74.17	−4.14	14.76	4.45	30.50	447.00	0.65	0.82	13.60
T	Rhodes	74.19	−4.54	15.25	4.19	14.00	240.00	0.25	0.48	14.45
MF	Ioannina	50.83	8.83	57.68	3.98	40.60	394.86	0.24	0.77	17.20
MF	Metsovo	62.59	−2.31	37.37	4.29	28.00	364.00	0.36	0.73	13.20
P	Thassos	69.56	−5.45	25.94	4.95	32.00	437.00	0.46	1.01	15.05
MF	Ioannina	51.08	8.95	58.12	4.32	41.20	324.00	0.25	0.55	18.90
CE	Arta	71.33	−3.04	13.35	4.81	23.39	294.09	0.87	0.61	17.90
F	Karditsa	72.94	−4.71	18.02	4.68	29.00	678.00	1.25	1.90	17.90
CE	Arta	77.08	−3.13	7.03	3.41	36.40	332.00	0.34	0.64	17.20
F	Metsovo	73.04	−3.85	16.71	4.72	26.50	645.00	0.87	1.25	17.60
	Mean	67.65^b^	−2.20^e^	27.42^g^	4.42^j^	28.60^l^	410.07^o^	0.53^r^	0.87^u^	16.26^x^
	±SD	8.36	5.01	16.18	0.44	7.69	126.38	0.30	0.37	1.83
	RSD_R_ %	0.62	31.82	2.04	0.63	9.79	0.68	5.28	2.60	1.72

	*Egypt*									
CL	GCA	76.14	−2.65	7.56	3.38	6.50	112.20	0.11	0.22	16.33
CL	GCA	75.88	−2.85	7.76	3.61	8.00	126.20	0.12	0.25	16.99
CL	GCA	76.05	−3.07	8.34	3.49	6.50	129.40	0.12	0.26	15.96
CL	GCA	76.72	−2.79	6.93	3.46	8.00	132.60	0.13	0.27	16.51
CL	GCA	75.70	−3.06	9.42	3.40	7.00	131.00	0.12	0.26	15.96
CL	GCA	76.69	−3.65	5.88	3.31	7.50	127.20	0.12	0.25	16.84
CL	GCA	76.45	−3.90	6.98	3.27	8.00	117.60	0.11	0.24	16.53
CL	GCA	75.88	−3.14	4.83	3.26	7.00	114.70	0.11	0.23	17.05
	Mean	76.19^c^	−3.14^e^	7.21^h^	3.40^k^	7.31^m^	123.86^p^	0.12^s^	0.25^v^	16.52^x^
	±SD	0.39	0.43	1.42	0.12	0.65	7.87	0.01	0.02	0.43
	RSD_R_ %	0.55	22.29	7.77	0.82	38.30	2.26	11.67	3.36	1.02

The reported results are the mean of three independent replicates (*n*=3), with the exception of colour parameters that are the mean of five independent replicates (*n*=5). Different lowercase letter in each column of the average value represents statistically significant differences for each physicochemical parameter according to the production area (*p* < 0.05). *N*: the number of honey samples; BO: botanical origin; GO: geographical origin; GCA: greater Cairo area; MF: multifloral; P: pine; F: fir; CE: citrus and erica; WT: wild thyme; T: thyme; CL: clover; FA: free acidity; TDS: total dissolved solids; EC: electrical conductivity; SD: standard deviation; RSD_R_ %: interlaboratory variation.

**Table 2:** Mineral content (mg/kg) of Cypriot, Greek, and Egyptian honeys.

*N*	BO	GO	Al	B	Ca	Zn	Si	Fe	P	Mn	Mg	Total minerals
		*Cyprus*										
1	MF	Larnaca	1.69	3.58	23.66	0.88	5.54	1.49	46.25	0.07	9.24	92.40
2	MF	Larnaca	1.63	5.14	93.11	1.10	4.94	0.96	65.77	0.91	18.62	192.18
3	MF	Larnaca	2.19	5.20	91.64	6.94	7.26	3.46	66.10	0.83	23.40	207.02
4	MF	Larnaca	0.49	4.11	54.10	1.61	6.35	1.74	53.95	0.10	20.27	142.72
5	MF	Limassol	1.46	2.12	30.21	1.09	4.94	1.45	40.06	0.08	14.12	95.53
6	MF	Limassol	1.02	3.22	48.34	1.62	5.61	1.52	56.09	0.15	13.05	130.62
7	MF	Limassol	0.53	2.56	49.76	1.93	5.34	1.43	54.26	0.19	11.31	127.31
8	MF	Limassol	1.46	3.35	35.66	0.99	4.69	1.35	51.56	0.27	10.73	110.06
9	MF	Limassol	1.71	4.42	60.88	0.91	6.83	1.77	75.69	0.23	15.59	168.03
10	MF	Limassol	1.60	4.48	58.63	0.93	7.05	1.65	54.16	0.48	12.16	141.14
11	MF	Nicosia	1.48	3.67	47.03	1.42	5.89	1.91	60.69	0.28	19.57	141.94
12	MF	Nicosia	1.77	4.14	69.21	3.77	6.68	3.52	53.62	0.42	13.49	156.62
13	MF	Nicosia	2.13	3.28	143.47	0.86	5.18	1.43	40.97	2.13	19.82	219.27
14	MF	Nicosia	24.43	2.17	27.89	1.55	7.28	3.97	257.19	4.11	80.48	409.07
		Mean	3.11^a^	3.67^c^	59.54^e^	1.83^h^	5.97^j^	1.98^m^	69.74^o^	0.73^r^	20.13^u^	166.70^x^
		±SD	6.16	0.98	32.13	1.65	0.92	0.94	54.81	1.11	17.88	79.60
		RSD (%)	1.98	0.27	0.54	0.90	0.15	0.47	0.79	1.52	0.89	

		*Greece*										
15	P	Rhodes	3.21	2.41	25.40	0.97	73.80	3.46	89.29	0.59	53.45	252.58
16	F	Messinia	27.35	2.84	15.22	1.24	8.18	4.16	217.33	4.37	109.53	390.22
17	P	Rhodes	2.93	2.67	23.63	9.30	73.63	3.22	92.67	0.57	53.96	262.58
18	WT	Lakonia	27.37	3.39	24.59	1.01	3.32	2.30	264.31	4.60	88.51	419.40
19	T	Rhodes	1.46	13.29	65.93	1.01	27.64	2.00	55.85	0.26	31.40	198.84
20	MF	Ioannina	9.15	4.72	59.88	3.52	9.58	2.81	138.80	5.54	82.72	316.72
21	MF	Metsovo	1.55	4.75	49.37	1.00	7.21	0.75	62.44	1.13	25.83	154.03
22	P	Thassos	0.40	4.49	37.18	1.48	7.09	0.89	105.75	2.19	26.28	185.75
23	MF	Ioannina	8.30	4.45	56.71	3.45	8.84	2.63	131.12	5.32	80.70	301.52
24	CE	Arta	7.75	1.69	36.07	2.53	96.42	4.11	170.02	2.01	60.20	380.80
25	F	Karditsa	3.28	1.33	30.32	1.20	11.17	1.18	57.25	0.54	20.13	126.40
26	CE	Arta	0.82	4.44	44.11	1.37	11.17	1.19	57.25	0.54	80.13	201.02
		Mean	7.80^b^	4.21^c^	39.03^f^	2.34^h^	28.17^k^	2.39^m^	120.17^p^	2.31^s^	59.40^v^	265.82^y^
		±SD	9.61	3.10	16.21	2.38	33.04	1.21	67.74	2.07	29.24	96.94
		RSD (%)	1.23	0.74	0.42	1.02	1.17	0.51	0.56	0.90	0.49	

		*Egypt*										
27	CL	GCA	1.21	0.65	82.23	1.09	9.09	8.45	26.23	0.28	17.49	146.72
28	CL	GCA	1.36	0.52	54.46	0.76	6.58	4.89	15.88	0.22	12.17	96.84
29	CL	GCA	2.07	0.78	77.07	0.55	0.80	2.18	26.33	0.17	18.39	128.34
30	CL	GCA	4.05	0.87	92.63	1.17	11.29	4.50	28.47	0.54	22.04	165.56
31	CL	GCA	0.95	1.40	112.10	0.78	11.54	6.58	39.47	0.55	21.42	194.79
32	CL	GCA	3.35	3.17	59.54	1.23	7.40	4.49	35.29	0.28	17.63	132.38
33	CL	GCA	13.06	2.69	44.79	1.68	5.36	4.82	26.55	0.20	15.24	114.39
34	CL	GCA	1.53	2.18	50.61	1.07	0.81	2.66	30.65	0.19	13.88	103.58
		Mean	3.45^a^	1.53^d^	71.68^g^	1.04^i^	6.61^l^	4.82^n^	28.61^q^	0.30^t^	17.28^w^	135.33^z^
		±SD	4.03	1.02	23.37	0.35	4.17	2.00	7.01	0.15	3.44	45.56
		RSD (%)	1.17	0.67	0.33	0.34	0.63	0.41	0.25	0.50	0.20	
		LOD	0.41	0.03	0.50	0.02	0.02	0.02	0.10	0.08	0.02	
		LOQ	1.34	0.10	0.17	0.07	0.07	0.07	0.33	0.24	0.07	

The reported results are the mean of three independent replicates (*n*=3). Different lowercase letter in each column of the average value represents statistically significant differences for each mineral according to the production area (*p* < 0.05). *N*: the number of honey samples; BO: botanical origin; GO: geographical origin; GCA: greater Cairo area; MF: multifloral; P: pine; F: fir; CE: citrus and erica; WT: wild thyme; T: thyme; CL: clover; FA: free acidity; TDS: total dissolved solids; EC: electrical conductivity; SD: standard deviation; RSD: relative standard deviation; LOD: limit of detection; LOQ: limit of quantification.

**Table 3:** Physicochemical parameters and minerals used for the botanical discrimination of Cypriot, Greek, and Egyptian honeys.

Discrimination parameters	Discriminant functions		*F*	*p*	Strong physicochemical markers of the provenience of the studied honey types
	1	2			
pH	0.585	0.297	34.979	<0.001	
EC	0.426^*∗∗*^	0.284	19.732	<0.001	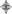
Moisture	−0.006	0.650	14.725	<0.001	
FA	0.458	−0.226^*∗∗*^	21.340	<0.001	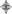
TDS	0.535^*∗∗*^	0.259	29.097	<0.001	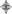
Salinity	0.347^*∗∗*^	−0.006	11.272	<0.001	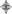
*L* ^*∗*^	−0.268^*∗∗*^	−0.103	7.059	0.003	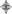
*b* ^*∗*^	0.318^*∗∗*^	0.121	9.942	<0.001	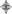
P	0.112^*∗∗*^	0.014	7.212	0.003	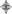
Fe	−0.092	−0.322	12.282	<0.001	
Ca	−0.086	0.012	4.289	0.023	
Mg	0.147	−0.163	14.381	<0.001	
Mn	0.098^*∗∗*^	−0.065	5.913	0.007	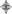
Si	0.082	−0.113^*∗∗*^	4.788	0.015	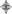
B	0.078	0.121^*∗∗*^	4.529	0.019	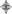

Pooled within-group correlations between discriminating variables and standardized canonical discriminant functions are given. Variables are ordered by the absolute size of correlation within function; ^*∗∗*^largest absolute correlation between each variable and any discriminant function (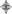); FA: free acidity; TDS: total dissolved solids; EC: electrical conductivity.
